# Supplementary material for: Cryopreserving Rabbit Semen: Impact of Varying Sperm Concentrations on Quality and the Standardization of Protocol
Source: Vet Sci. 2023 Dec 22;11(1):9. doi: 10.3390/vetsci11010009 (PMC10818829; doi:10.3390/vetsci11010009)
Supplement: Supplementary file 1 [file vetsci-11-00009-s001.zip › vetsci-2737584-supplementary.pdf]

Supplementary materials

Table S1. Fresh sperm quality outcomes in different rabbit semen pools.

	Sperm variables											
**Fresh semen pool**	**Sperm concentration (spz × 10^6/mL)**	**TM (%)**	**PM (%)**	**VCL (µm/sec)**	**VAP (µm/sec)**	**VSL (µm/sec)**	**STR (%)**	**LIN (%)**	**WOB (%)**	**ALH (µm)**	**BCF (Hz)**	**SMI (%)**
**1**	765	95.1	68.8	73.9	39.5	23.1	57.5	32.2	53.8	3.3	6.9	85.7
**2**	655	95.2	67.3	73.3	39.9	26.2	59.7	34.0	53.4	3.3	6.8	94.1
**3**	639	89.6	68.9	75.5	43.9	32.3	68.3	41.8	58.1	3.1	8.4	89.7
**4**	1,105	92.9	76.5	87.1	40.1	26.1	62.3	30.4	47.4	4.1	8.3	92.5
**5**	672	92.3	70.6	76.9	42.8	29.0	64.2	37.6	55.6	3.4	7.7	92.5
**6**	746	88.2	65.5	75.2	40.1	24.6	59.7	33.5	53.7	3.4	7.6	88.0
**7**	628	89.5	66.5	70.8	35.6	21.2	57.2	29.3	49.8	3.3	7.1	90.4
**8**	650	91.1	61.9	66.2	37.6	24.4	60.5	36.4	56.9	3.0	7.1	90.2
**Mean**	**732.5**	**91.7**	**68.2**	**74.9**	**39.9**	**25.9**	**61.2**	**34.4**	**53.6**	**3.4**	**7.5**	**90.4**
**S.D.**	**158.6**	**2.6**	**4.2**	**6.0**	**2.6**	**3.5**	**3.7**	**4.1**	**3.5**	**0.3**	**0.6**	**2.7**

TM (%), total motility; PM (%) progressive motility; VCL (µm/sec), curvilinear velocity; VAP (µm/sec), average path velocity; VSL (µm/sec), straight-line velocity; STR (%) (VSL/VAP × 100), straightness; LIN (%) (VSL/VCL × 100), linearity; WOB (%) (VAP/VCL × 100), wobble; ALH (µm), amplitude of lateral head displacement; BCF (Hz), beat cross frequency; SMI (%) sperm membrane integrity.
